# Acetone Sensor Based on FAIMS-MEMS

**DOI:** 10.3390/mi12121531

**Published:** 2021-12-09

**Authors:** Junna Zhang, Cheng Lei, Ting Liang, Ruifang Liu, Zhujie Zhao, Lei Qi, Abdul Ghaffar, Jijun Xiong

**Affiliations:** 1 State Key Laboratory of Dynamic Measurement Technology, North University of China, Taiyuan 030051, China; nuczjn@163.com (J.Z.); s2006222@st.nuc.edu.cn (Z.Z.); xiongjijun@nuc.edu.cn (J.X.); 2 State Key Laboratory of Bioelectronics, Southeast University, Nanjing 210096, China; lrfnuc@163.com; 3 North Automatic Control Technology Institute, Taiyuan 030006, China; 18635113272@163.com; 4 State Key Laboratory of Geomechanics and Geotechnical Engineering, Institute of Rock and Soil Mechanics, Chinese Academy of Sciences, Wuhan 430071, China; 92ghaffar@gmail.com; 5 University of Chinese Academy of Sciences, Beijing 100049, China

**Keywords:** diabetes, FAIMS-MEMS, acetone, reproducibility, stability

## Abstract

In this paper, to address the problems of large blood draws, long testing times, and the inability to achieve dynamic detection of invasive testing for diabetes, stemming from the principle that type 1 diabetic patients exhale significantly higher levels of acetone than normal people, a FAIMS-MEMS gas sensor was designed to detect acetone, which utilizes the characteristics of high sensitivity, fast response, and non-invasive operation. It is prepared by MEMS processes, such as photolithography, etching, and sputtering, its specific dimensions are 4000 μm in length, 3000 μm in width and 800 μm in height and the related test system was built to detect acetone gas. The test results show that when acetone below 0.8 ppm is introduced, the voltage value detected by the sensor basically does not change, while when acetone gas exceeds 1.8 ppm, the voltage value detected by the sensor increases significantly. The detection accuracy of the sensor prepared by this method is about 0.02 ppm/mV, and the voltage change can reach 1 V with a response time of 3 s and a recovery time of 4 s when tested under 20 ppm acetone environment; this has good repeatability and stability, and has great prospects in the field of non-invasive detection of type 1 diabetes.

## 1. Introduction

Diabetes mellitus (DM) is a metabolic syndrome of fasting and postprandial hyperglycemia caused by inadequate insulin secretion. It is a syndrome that may be due to inadequate insulin secretion or defective insulin action, leading mainly to disorders of sugar, protein, and lipid metabolism. Currently, it is the third major non-communicable disease threatening human health, after cerebrovascular diseases and tumors—from the International Diabetes Federation (IDF). The key data on diabetes in China are: in 2019, China had about 116.4 million people with diabetes (20–79 years old), China has the highest number of people with diabetes in the world, accounting for 25% of the world’s diabetic population. A total of 1 in 9 adults (20–79 years old) have diabetes. In 2019, about 4.2 million people died from diabetes or its complications worldwide, which is equivalent to 1 death every 8 s. It accounts for about 11.3% of global deaths due to diabetes and is still on the rise in the coming years. There are two common forms of diabetes, type 1 diabetes, which is characterized by early onset and the inability to produce insulin, and type 2 diabetes, which is characterized by late onset and the inability to properly utilize insulin [1]. Currently, blood glucose is usually measured clinically with a blood glucose meter, and blood samples are collected mainly by the needle prick method, followed by qualitative analysis with disposable test strips. However, patients are usually resistant to this method because it causes damage to skin tissues and is accompanied by an obvious stinging sensation, and there are unsafe factors that can easily cause infection. Therefore, the development of a noninvasive detection technique has become a hot spot in medical research, and breath analysis, as a new noninvasive detection technique, has been favored by many researchers [2,3,4,5,6,7,8]. Studies have shown that the human breath contains more than 1000 volatile organic compounds (VOCs) [9]. Acetone is the second highest VOC in human breath, and medical researchers have shown that the concentration of acetone exhaled by normal individuals is less than 0.8 ppm, while the concentration of acetone exhaled by patients with type 1 diabetes is higher than 1.8 ppm; therefore, real-time monitoring of acetone in human breath can help in the prevention and diagnosis of type 1 diabetes [10,11,12,13,14,15,16,17].

The main sensors for VOC detection are electrochemical gas sensors and metal oxide gas sensors. Electrochemical gas sensors [18,19] are small, inexpensive, and sensitive, but the disadvantages are poor selectivity, a high concentration of gas components leading to permanent sensor failure, and the recovery time after each measurement of the sensor, which takes tens or even hundreds of seconds. Metal oxide gas sensors [20] are inexpensive but have poor stability, are subject to environmental influences, and the structure is not easy to miniaturize. High-field asymmetric waveform ion mobility spectrometry (FAIMS) [21] is a technique that works by exploiting the difference in ion mobility at high and low electric fields, and has the advantages of small size, high sensitivity, wide detection range, and fast response. FAIMS has great advantages in detecting VOCs and can effectively select target chemicals [22]. The components of FAIMS technology can be the MEMS technology to achieve miniaturization and increase sensitivity and resolution. Breath analysis is used as an adjunct to the diagnosis and monitoring of type 1 diabetes, but non-invasive analysis of breath for disease detection using techniques, such as proton transfer reaction mass spectrometry (PTR-MS), gas chromatography coupled with mass spectrometry (GC-MS), and selective ion flow tube mass spectrometry (SIFT-MS) has long sampling times, lacks specificity, is bulky, requires trained operators who need to test identification [1], has long test analysis times, and point-of-care-based sensor technologies “electronic noses” (eNose) often have stability and sensitivity problems [22]. Therefore, there is an urgent need to develop a sensor that is portable, simple to operate, and has a short analysis time. This requires the use of microelectromechanical systems (MEMS) technology. There are many sensors on the market that take advantage of this technology to achieve miniaturization, low cost, and the mass production of sensors. One of the most popular sensors is the MEMS inertial sensor, which is processed and manufactured using MEMS technology [23]. Inertial sensors include accelerometers and angular velocity sensors (gyros) and their single, dual, and triaxial combinations IMU (inertial measurement unit) and AHRS (attitude reference system including magnetic sensors), where MEMS accelerometers are sensors for inertial force measurement using sensing masses. These usually consist of standard mass blocks (sensing elements) and detection circuits. Commonly used inertial sensors are accelerometers and gyroscopes, which usually enable acceleration measurement, tilt measurement, vibration measurement, and even rotation measurement, they are commonly used in consumer electronics products [24], but cannot meet our measurement needs for gases. FAIMS sensors detect specific substance molecules, which enable smoke detection, battlefield chemical warfare agent detection, human exhaled gas detection, toxic gases, trace detection of volatile organic compounds (such as water organic pollutants), miniaturization of sensors using MEMS technology processing, and high sensitivity. In short, FAIMS-MEMS sensors achieve rapid identification of gases on site and in situ detection. They are simple and miniaturized equipment, and are convenient and portable [25,26,27,28].

Therefore, we designed a sensitive head with an integrated parallel migration zone collection area consisting of upper and lower polar plates using the FAIMS principle, and the sensitive head was made by MEMS technology, such as photolithography, etching, and sputtering, its specific dimensions are 4000 μm in length, 3000 μm in width and 800 μm in height. In the experiment, after applying high field asymmetry voltage and compensation voltage to the electrode corresponding to the sensitive head migration region, the screening of specific ions was achieved. Finally, the gas concentration is measured by voltage detection of the detection zone. The experimental results show that the sensor prepared by this method can exclude the interference of nitrogen and moisture, as well as achieve the specific detection of acetone; therefore, it has great application prospects in the field of noninvasive detection technology for type 1 diabetes [22].

## 2. The Basic Working Principle of FAIMS

### 2.1. Principle and Sensor Design

**Figure 1:**
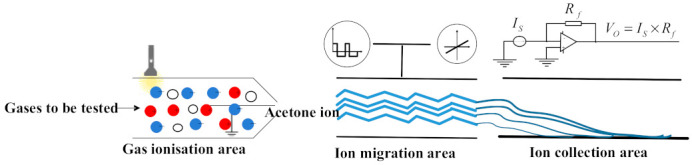
The structure of the FAIMS gas sensor.

Principle: The structure of the FAIMS gas sensor consists of three main parts: the ionization zone, the migration zone, and the collection zone. The structure of the FAIMS gas sensor is shown in Figure 1. The gas to be detected is ionized into positively or negatively charged ions in the ionization zone; the ions in the migration zone are passed through a high field asymmetric voltage and a DC compensating voltage, and the level of the ions to be detected passes through the migration zone to the collection zone, while other ions collide with the pole plate in the migration zone; the collection zone is used for voltage detection to achieve the detection of different gas concentrations.

#### 2.1.1. The Ionization Area

The ions need to be dissociated from the gas molecules before they can enter the migration zone.

#### 2.1.2. The Migration Zone

**Figure 2:**
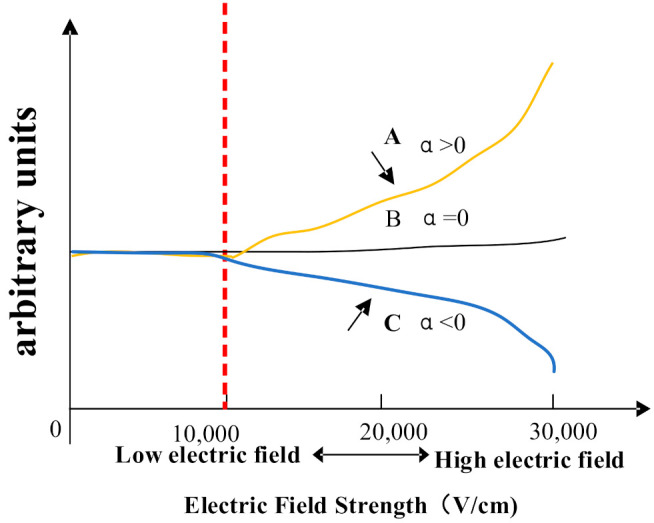
The variation curves of ion mobility at high and low electric fields [21].

The migration zone is the key part of the FAIMS gas sensor, which is based on the principle that the mobility of ions differs in high and low electric fields, as shown in the figure below. At electric field strengths below 11,000 V/cm, the electric field strength remains almost constant, while when the electric field strength is greater than 11,000 V/cm, the mobility coefficient of ions changes nonlinearly with the field strength, corresponding to different variation curves for each substance [29]. Figure 2 shows the variation curves of ion mobility at high and low electric fields. There are three main types of ions: A, B, and C, where α is the ion mobility coefficient; the ion mobility increases, stays the same, and decreases with increasing electric field strength.

**Figure 3:**
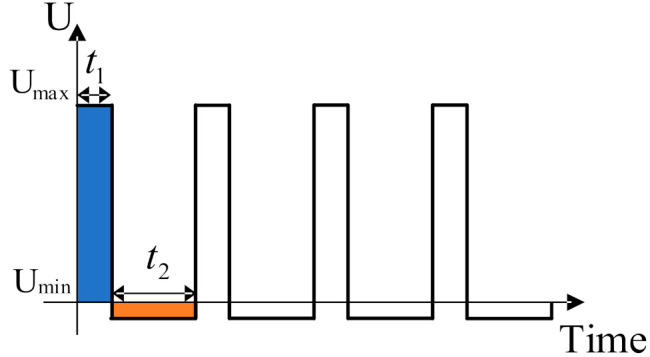
**The** High field asymmetric voltage waveform diagram.

**Figure 4:**
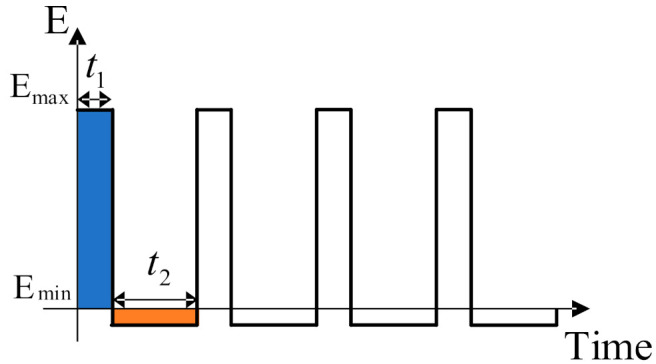
The high field asymmetric field strong waveform diagram.

When a gas is ionized into ions passing through the migration zone, it will move directionally under the action of high and low electric fields. Figure 3 below shows the high field asymmetric voltage waveform diagram, where U_max_ and U_min_ are expressed as the maximum and the minimum voltage [30]. As the distance between the pole plates is certain, the maximum electric field strength generated at the maximum voltage is E_1_. The minimum electric field strength generated at the minimum voltage is E_2_. The high field asymmetric field strong waveform diagram is shown in Figure 4.

The electric field strength of the ion in the high field is E_1_. The time of motion is t_1_, the electric field strength of the ion in the low field is E_2_, and the time of motion is t_2_; E_1_t_1_ = E_2_t_2_. It is assumed that the ion’s mobility in the high field is K_1_, and the mobility in the low field is K_2_ and is a negative ion. Within an asymmetric voltage waveform, the displacement of the ion in the high field is S_1_, and the ion produced in the low field is displacement S_2_ and has Equations (1) and (2).

(1) $$S_{1}=K_{1}E_{1}t_{1}$$

(2) $$S_{2}=K_{2}E_{2}t_{2}.$$

If K_1_ > K_2_, then the ion will be shifted a certain distance in the direction of the high electric field, and the distance shifted in a single cycle can be shown in Equation (3).

(3) $$\Delta S=S_{1}-S_{2}=K_{1}E_{1}t_{1}-K_{2}E_{2}t_{2},$$

**Figure 5:**
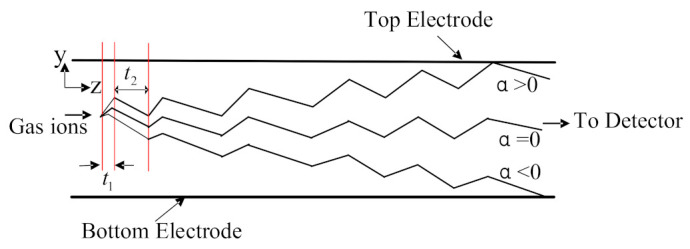
The trajectory of ions in an asymmetric high and low electric field.

**Figure 6:**
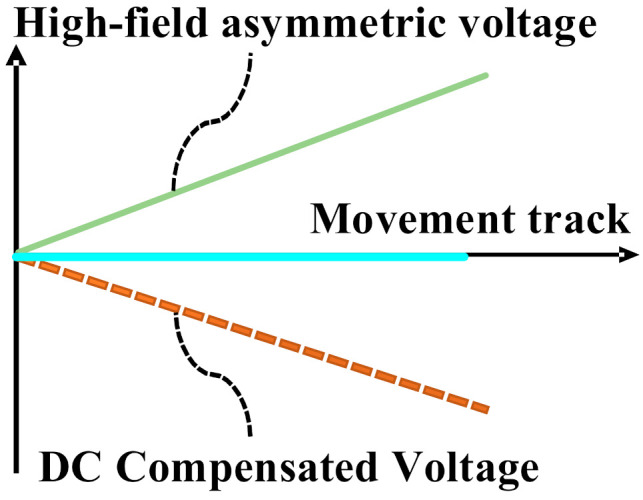
The trajectory of the ions under the action of the compensation voltage and high asymmetric voltage.

The ions move in the high field asymmetric voltage, the ion mobility is different in high and low fields, and the ions will have a deviation of upward or downward displacement in each cycle. Meanwhile, the applied high field asymmetric voltage has a high frequency; therefore, the superposition of multiple cycles of displacement in the migration zone will eventually lead to the collision between the ions and the pole plate. Below, Figure 5 shows the trajectory of ions in an asymmetric high and low electric field [29], which require the application of a specific compensation voltage; eventually, the ions move to the electrode plate in the collection area under the action of the compensation voltage, and Figure 6 below shows the trajectory of the ions under the action of the compensation voltage and high asymmetric voltage.

#### 2.1.3. The Detection Area

The ions move to the detection area and build the relevant circuit for detection. The detection area adopts the micro-current detection method: the charged ions move to the pole plate of the detection area, forming a weak current on the pole plate, and the current of PA magnitude is amplified to the voltage of MA magnitude through an external amplifier circuit to achieve the detection of the concentration.

### 2.2. Design of the Sensor

#### 2.2.1. The Ionization Zone

Before the ions can enter the migration zone, the gas molecules need to be ionized. In the experiments, the chosen ionization source is a 10.6 eV vacuum mercury lamp (Heraues). The main theory is that the target gas is bombarded by UV light generated by the ion lamp, which absorbs a certain amount of UV energy and then ionizes, i.e., ionizes [29], as shown below, in Equation (4).

(4) $$hv+M\to M^{+}+e$$

**Figure 7:**
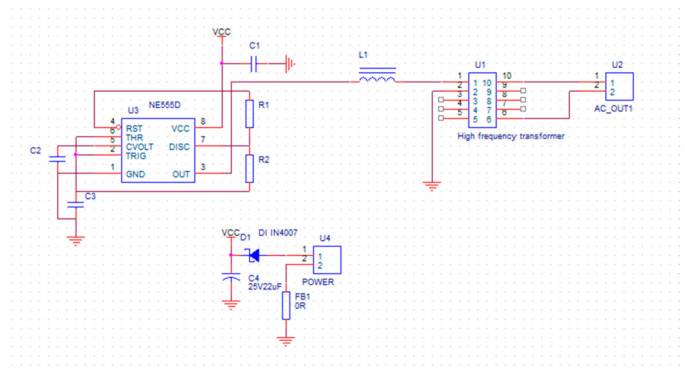
The circuit schematic of the ionization zone.

The ionization energy of the acetone gas molecule is 9.43 eV; therefore, when acetone gas passes through this ionization lamp, it will be ionized. Below, Figure 7 shows the circuit schematic of the ionization zone.

#### 2.2.2. The Migration Area and the Collection Area

Voltage breakdown theory: Paschen’s law is used to describe the law of gas breakdown voltage for parallel pole plates. In order to assure that no breakdown occurs between the top and bottom pole plates when high field strength conditions are applied, it is necessary to confine the magnitude of the applied voltage, as in Equation (5), which is a function of the gas pressure and the parallel pole plates [30].

(5) $$V=\frac{apd}{In(pd)+b}$$

where *V* denotes breakdown voltage, *p* denotes gas pressure, and *d* denotes electrode plate spacing. In addition, the constants, *a* and *b*, are related to the type of carrier gas between the electrode plates. At standard atmospheric pressure *a* = 43.6 × 10^6^ V/(atm‧m) and *b* = 12.8.

When moving in the migration zone, the ions need a high electric field range: 10 KV/cm–30 KV/cm, the pole plate spacing directly influences the applied voltage strength; in the experiments, the pole plate spacing is chosen to be 50 μm. If the air is used as the carrier of the reaction gas and the test is carried out at room temperature, and standard atmospheric pressure, the breakdown voltage size can be calculated according to Equation (5) to be 754 V. In order to ensure that high field strength is achieved, the voltage value that needs to be applied for a 50 μm pole plate spacing is 50–150 V, which is much smaller than the breakdown voltage.

In addition, from the definition of deformation theory, when the voltage is applied to the pole plate, it will cause the plate to deform, especially under the action of a high-frequency electric field. This may cause the plate to shake and even fracture in serious cases. The maximum deformation of the plate is analyzed, and the formula is shown in (6).

(6) $$Y=\frac{\varepsilon E^{2}L^{4}}{64Ph^{3}}$$

where *Y* denotes the maximum deformation of the electrode plate; *ε* is the dielectric constant of the gas between the electrode plates in the ion migration zone, generally air. *L* means the length of the electrode plate in the migration zone; *P* means the Young’s modulus of the electrode plate material (BF33 glass and silicon wafer), and h is the thickness of the electrode plate, which can be equated to the thickness of the substrate.

Based on the above design principles, in this paper, a FAIMS-MEMS gas sensor is designed with an external ionization lamp for the ionization region and an integrated mems process for the migration and collection regions. With a field strength range of 10 KV/cm–30 KV/cm for ions, in order to minimize the applied voltage and the difficulty of the manufacturing process, the length of the pole plate in the migration area is 2000 μm, the width is 1000 μm, the pole plate spacing is 50 μm, the length of the pole plate in the collection area is 1000 μm, the width is 1000 μm, and the spacing between the migration area and the detection area is 50 μm. It can be calculated that the deformation caused by the pole plate of the silicon wafer is 0.1949 μm, and the deformation caused by the glass wafer is 0.1111 μm. The deformation is very small.

#### 2.2.3. Readout Circuit

**Figure 8:**
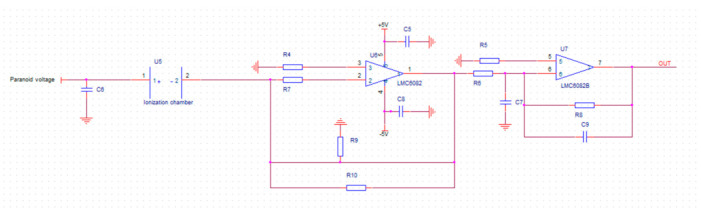
The schematic diagram of the readout circuit.

The readout circuit uses the current detection method: charged ions move to the pole plate in the detection area, forming a current in the pole plate, and converting the current to voltage through an external circuit to achieve the detection of the concentration. Figure 8 below shows the schematic diagram of the readout circuit.

### 2.3. Manufacturing of Sensors

**Figure 9:**
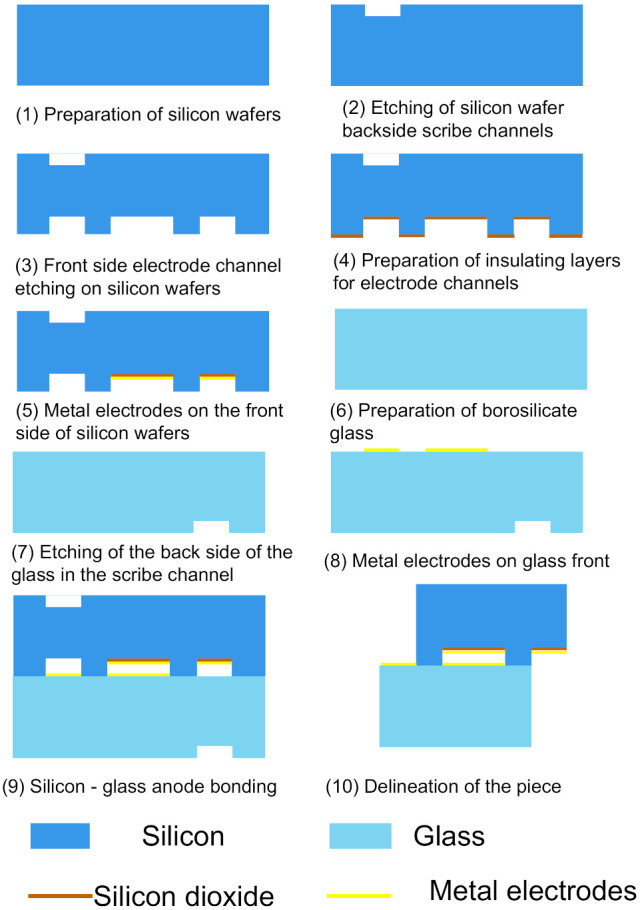
The schematic of each step of the sensor fabrication work.

**Figure 10:**
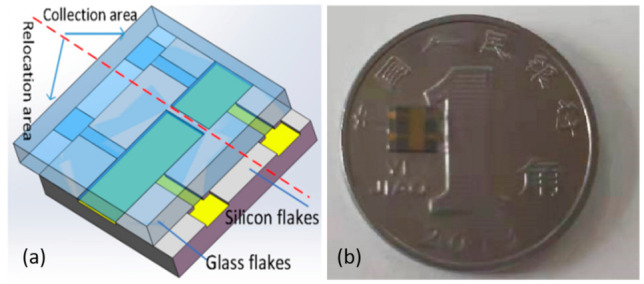
(**a**) the three-dimensional structure of the sensor (**b**) the physical view of the sensor.

**Figure 11:**
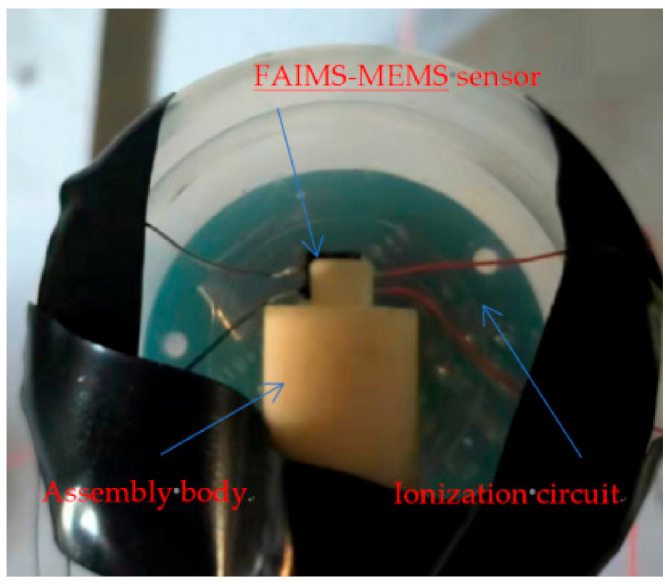
The assembly diagram of the ionization circuit and FAIMS-MEMS sensor.

The above-designed photoreceptor is prepared by the MEMS process, which mainly includes silicon wafer structure preparation, glass structure preparation, and silicon–glass anode bonding. The specific process flow is as follows: (1) Cleaning of silicon wafers—double-throw silicon wafers with crystal orientation of <100> and thickness of 300 μm (Suzhou Research Material Micro-Nano Processing Center) were selected for experiments, and SPM cleaning and RCA cleaning were performed to remove the organic staining on the silicon wafer surface and the natural oxide layer on the silicon wafer surface; (2) Scratch etching on the back side of the wafer. Etch a 5000 Å scribe slot on the backside of the wafer with an RIE etcher; (3) Etch the electrode slot on the front side of the wafer. A 50 µm electrode slot is etched on the front side of the wafer by a deep silicon etching process; (4) preparation of the electrode channel insulation layer—to maintain the insulation between the silicon wafer and the metal electrode, a silicon dioxide insulation layer is deposited on the electrode channel prepared in step (3) by PECVD technology and the insulation layer is patterned by a wet etching process; (5) metal electrode on the front side of the wafer. The TI attachment layer and AU metal layer are sputtered in the insulating layer area prepared in (4) by magnetron sputtering technology to realize the preparation of the silicon wafer electrode plate. (6) Cleaning of borosilicate glass: borosilicate glass with a thickness of 500 μm was selected for the experiment (Beijing GIN KOO MEMS Scientific & Technological Co., Ltd., Beijing, China), and the same SPM cleaning and RCA cleaning were performed to remove organic stains on the surface; (7) etching of scribing grooves on the back side of the glass: the glass was etched with an RIE etching machine to form 5000 Å scribing grooves on the backside of the glass; (8) metal electrodes on the front side of the glass. The metal electrode on the glass front is prepared by magnetron sputtering technique and the pattern of the metal electrode is made by the peeling technique; (9) silicon-glass anode bonding: Prepared silicon wafers are aligned with glass wafers and then bonded by increasing the temperature of the wafer-glass pair and adding an electrical potential across the wafer pair; (10) scribing—the prepared silicon wafers are mechanically cut into individual small chips. Below, Figure 9 shows the schematic of each step of the sensor fabrication work, and Figure 10 shows the three-dimensional structure of the sensor and the physical view of the sensor. Figure 11 shows the assembly diagram of the ionization circuit and FAIMS-MEMS sensor.

## 3. Results and Discussion

### 3.1. Test Systems

**Figure 12:**
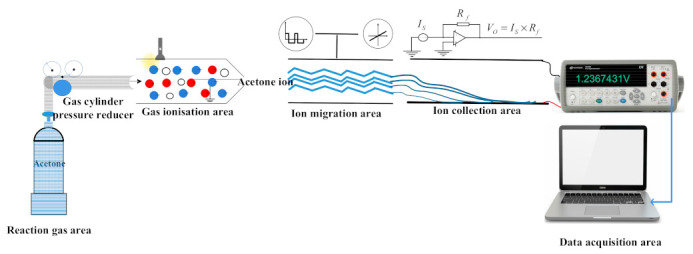
The schematic diagram of the test system.

**Figure 13:**
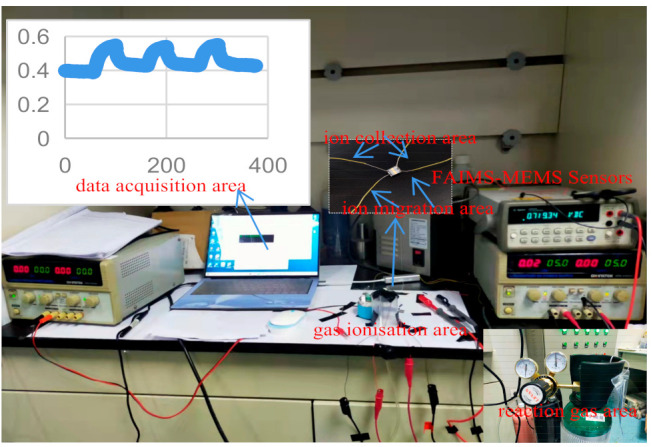
The physical diagram of the test system.

The complete test system mainly consists of reaction gas area, gas ionization area, ion migration area, ion collection area, and data acquisition area. The primary purpose of the gas reaction area is to provide the reaction chamber with the relevant concentration of acetone gas, which is mainly achieved by introducing different concentrations of standard acetone gas into the closed vessel. The gases tested in this experiment are all standard gases provided by Taiyuan Tainan Gas Co., (Shanxi, China) the gases are all single substances of volatile organic compounds, and the flow of these gases is kept constant by the cylinder pressure reducer; the ionization area uses a photo-ionization detector (PID), and the ionization lamp uses a mercury lamp with the energy of 10.6 eV. Ion migration regions and ion collection regions were prepared using the MEMS process and were connected to the corresponding circuit for ions to reach the detection region for detection; the data collection area is used to collect data via a high accuracy digital multimeter and the corresponding computer. The schematic diagram of the test system is shown in Figure 12. The physical diagram of the test system is shown in Figure 13.

### 3.2. Testing of Sensors

In the experiment, the sensitive head and the ionization device are placed in an airtight environment. It is necessary to ensure that the temperature and humidity are the same for each test. The test is carried out by controlling the concentration of acetone in the airtight container. At the same time, to ensure the accuracy of the test, dry air needs to be introduced for 10 min before each test so that the whole test system is in a more stable state.

#### 3.2.1. Testing of Acetone Gas

In order to verify the selectivity of the sensor for acetone gas detection applied to type 1 diabetics, a number of comparative experiments were performed: 0.8 ppm, 1.8 ppm, 10 ppm, and 20 ppm concentrations of acetone standard gas were tested. Table 1 shows the different gas concentrations corresponding to the different cycles in the experiments. The measurement method of this gas sensor is the relative comparison method: firstly, the instrument is calibrated with zero gas and standard concentration of gas, and the standard curve is stored in the instrument. During the measurement process, the instrument compares the electrical signal generated by the gas concentration to be measured with the electrical signal of the standard concentration, so as to calculate the accurate gas concentration value.

**Figure 14:**
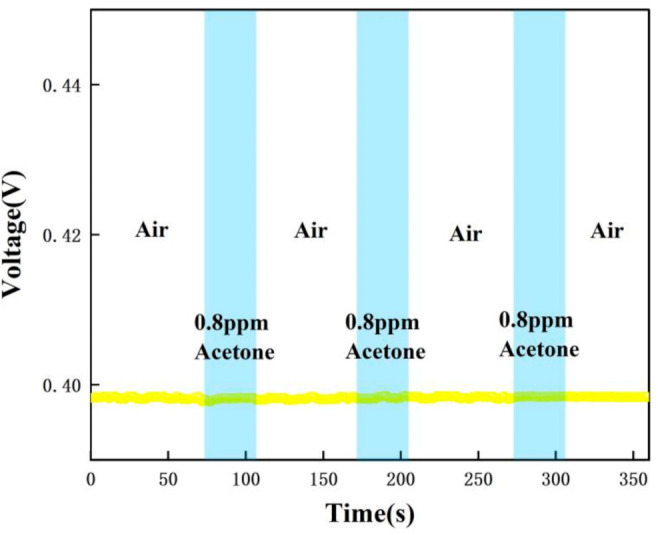
Test result of 0.8 ppm acetone.

**Figure 15:**
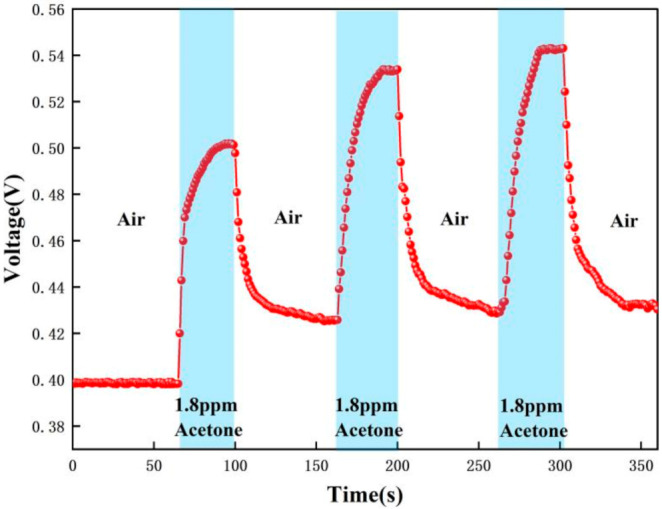
Test result of 1.8 ppm acetone.

**Figure 16:**
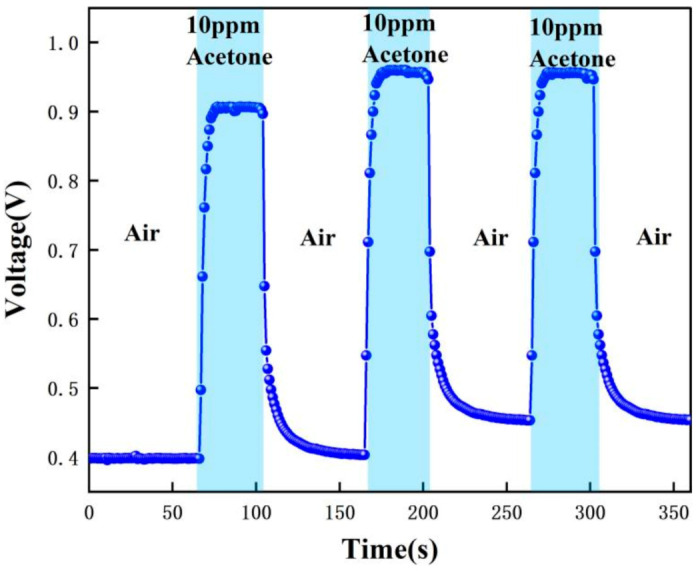
Test result of 10 ppm acetone.

**Figure 17:**
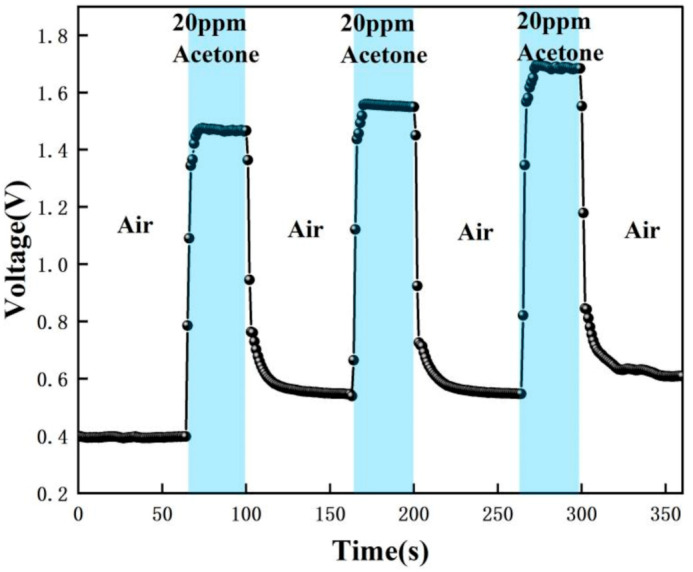
Test result of 20 ppm acetone.

The test data were recorded by computer software and the response graphs were plotted as shown in Figure 14, Figure 15, Figure 16 and Figure 17. As shown in Figure 14, at the maximum concentration of acetone exhaled by a normal person (0.8 ppm acetone) the corresponding voltage was 0.398 V, and the original voltage of the sensor into the circuit is 0.398V, so the corresponding voltage value varied by about 0 V, which was essentially constant in air. Due to the special detection circuit, the weak current generated by the ionization of the gas into ions of 0.8 ppm and below 0.8 ppm is covered by the noise of the circuit; this variation cannot be detected. As can be seen in Figure 15, the corresponding voltage in the acetone environment at 1.8 ppm is 0.5 V, with a corresponding voltage change of 102 mV. As can be seen in Figure 16, the corresponding voltage under the passage of 10 ppm acetone is 0.9 V, with a voltage change of 502 mV. As can be seen in Figure 17 below, the pass-through of 20 ppm acetone corresponds to a voltage of 1.4 V and a voltage change of 1002 mV.

**Figure 18:**
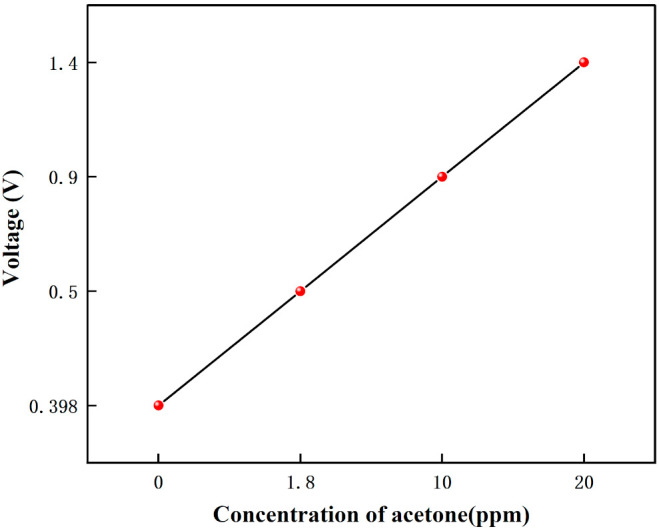
The voltage response graph for different concentrations of acetone.

**Figure 19:**
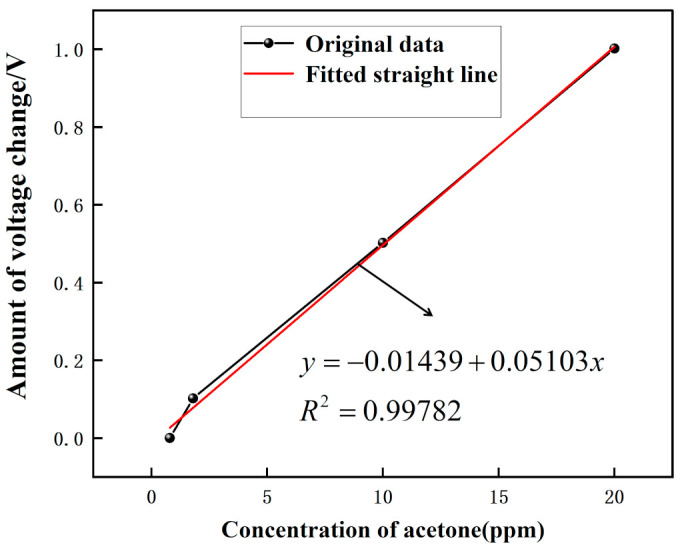
The voltage variation graph for different acetone concentration environments.

Figure 18 shows the voltage response graph for different concentrations of acetone and the results are linear. The voltage variation graph for different acetone concentration environments are shown in Figure 19. The range of 0–20 ppm of acetone gas environment shows a linear trend with increasing concentration, and the linearity is 99.782%. Sensor sensitivity of approximately 0.05103 V/ppm. This experiment shows that when no acetone passes, the voltage is 0.398 V; when 1.8 ppm acetone passes, the voltage is 0.5 V; when 10 ppm acetone passes, the voltage is 0.9 V; and when 20 ppm acetone passes, the voltage is 1.4 V. By calculation, we can get that when the gas passes. The voltage rise change is 0.102 V, 1.8 ppm of acetone is detected; when the voltage rise change is 0.502 V, 10 ppm of acetone is detected; and when the voltage rise change is 1.002 V, 20 ppm of acetone is detected. The sensor can detect acetone gas with an accuracy of about 0.02 ppm/mV.

**Figure 20:**
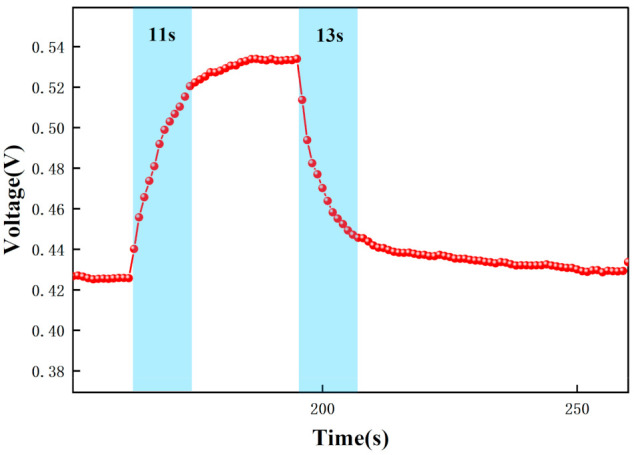
Response recovery time for testing at 1.8 ppm acetone.

**Figure 21:**
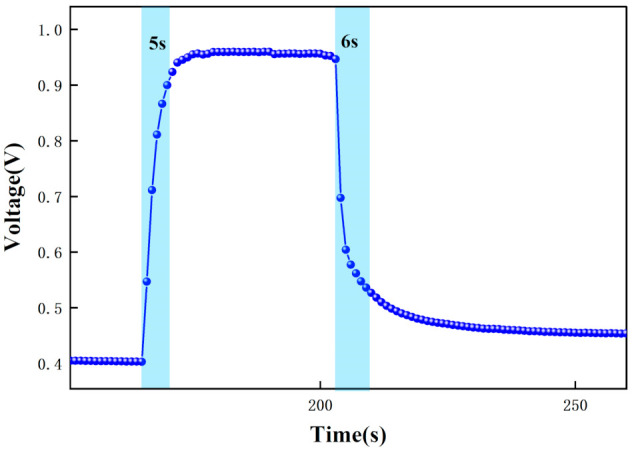
Response recovery time for testing at 10 ppm acetone.

**Figure 22:**
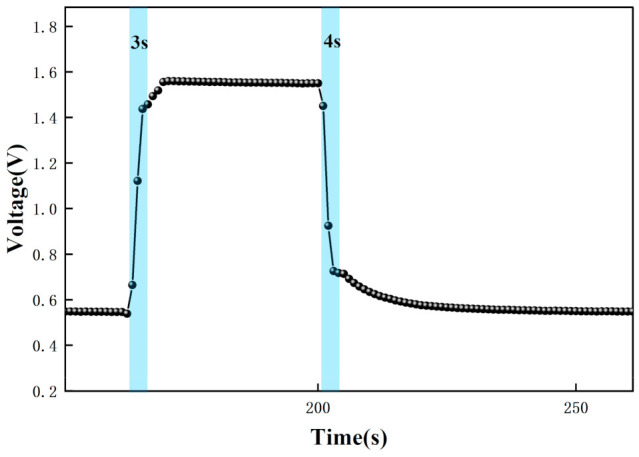
Response recovery time for testing at 20 ppm acetone.

In addition, the response time and recovery time of the sensors were tested at different concentrations. The response time and recovery time are the times required for the sensor to reach a 90% response and recovery [2]. Figure 20, Figure 21 and Figure 22 show the response/recovery time plots for 1.8, 10, and 20 ppm concentration environments, respectively (Selected from Figure 15 to Figure 17 for the 151–260s time). Figure 20 shows a sensor response time of 11 s and a recovery time of 13 s for the 1.8 ppm acetone environment. Figure 21 shows a response time of 5 s and a recovery time of 6 s in a 10 pm acetone environment, and Figure 22 shows the response time of 3s and the recovery time of 4s at 20 ppm acetone. The test results show that the response of the sensor is different in different concentrations of the acetone environment; with the increase of gas concentration, the response value becomes larger and the response time and recovery time become shorter. This is because the higher the concentration of acetone, the more acetone molecules pass through the ionization zone, i.e., the more are ionized, and after that, through the migration zone, the more ions reach the detection zone, and thus the voltage value of the detection zone further changes.

**Figure 23:**
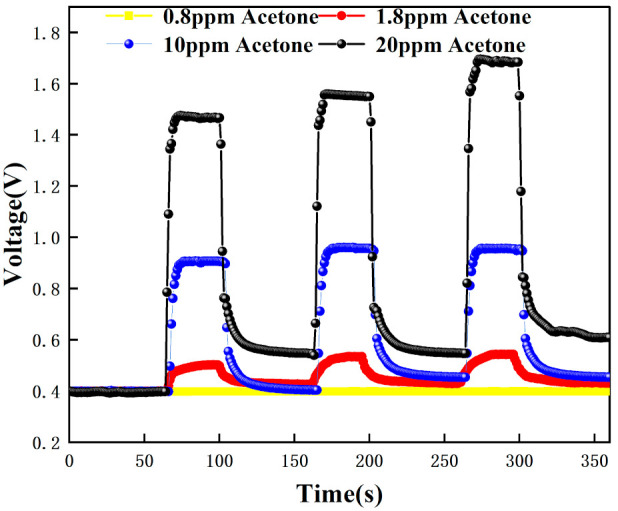
Response test results for different concentrations of acetone.

Figure 23 below compares the responses for the four cases of 0.8 ppm, 1.8 ppm, 10 ppm, and 20 ppm. It can be clearly found that for 0.8 ppm there is no change in voltage, while for 1.8–20 ppm there is a significant change in voltage. The higher the concentration of acetone, the greater the change in voltage. Therefore, based on the fact that the level of exhaled acetone is significantly higher in type 1 diabetic patients than in normal patients, noninvasive monitoring of type 1 diabetic patients can be achieved with our sensor. In addition, the response curve of the sensor is consistent after several tests, all of which can be recovered to a stable value with good repeatability and stability.

Figure 15, Figure 16, Figure 17, Figure 18, Figure 19, Figure 20, Figure 21, Figure 22 and Figure 23: The reason for the baseline change is the normal minimal fluctuation of the system circuit itself. The high precision multimeter and its matching AG34401A software show high accuracy, and the data can be collected to nv level. The baseline change is the change of nv level data, which is a normal phenomenon in circuit detection and is unchangeable.

#### 3.2.2. Nitrogen Interference Test

**Figure 24:**
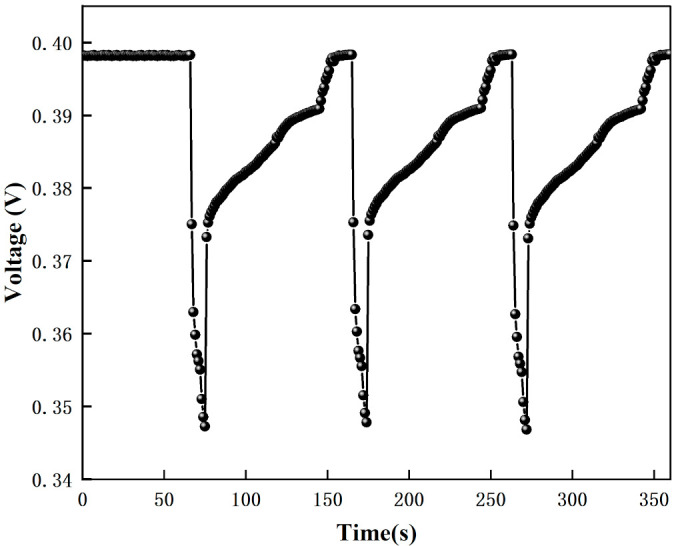
The test results of nitrogen.

The human body exhales more than 2500 kinds of gas components; it is impossible to test all impurity gases one by one. Human-exhaled gas components are mainly nitrogen, and nitrogen (99.999% purity) was tested. Figure 24 shows the test results of nitrogen. Figure 24 shows that the voltage instantly decreases when nitrogen is passed in, and the voltage returns to the initial value when the nitrogen is stopped. This is due to the fact that the ionization energy of nitrogen is 15.5808 eV, which is greater than the energy of the UV lamp (10.6 eV), and nitrogen cannot be ionized. The change in voltage when nitrogen is introduced is due to the change in pressure in the test chamber. Through the nitrogen voltage decreases and through the acetone voltage remains unchanged or increases, excluding the effect of nitrogen on acetone measurement.

**Figure 25:**
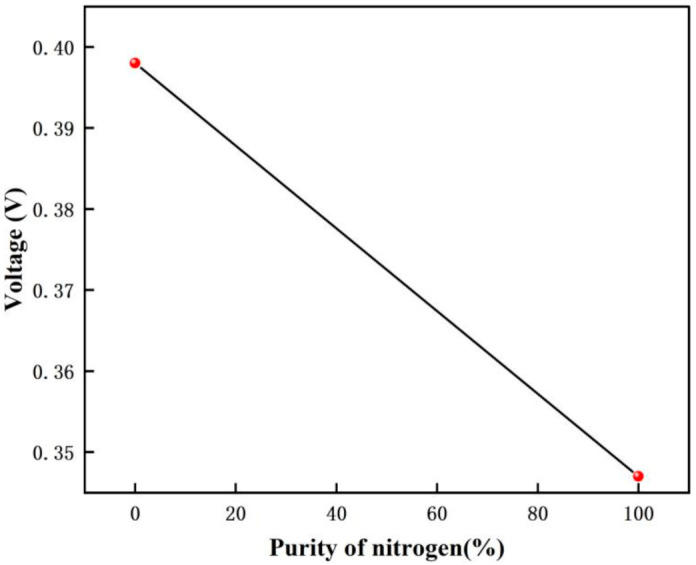
The voltage response graph for different purity nitrogen environments.

**Figure 26:**
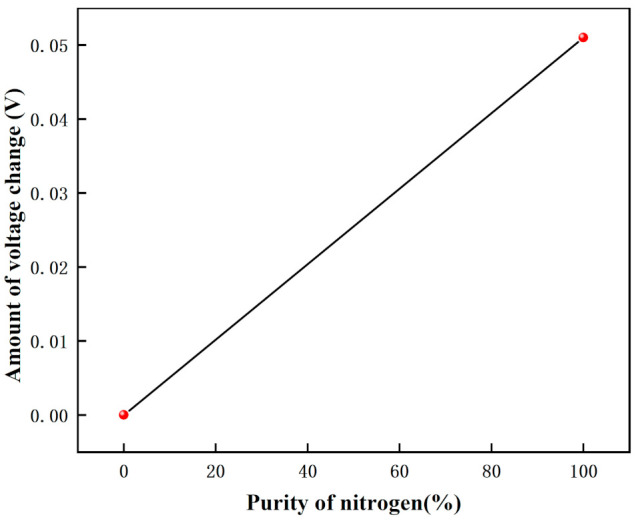
The voltage variation graph for different purity nitrogen environments.

The voltage response graph for different purity nitrogen environments are shown in Figure 25, and the voltage variation graph for different purity nitrogen environments are shown in Figure 26. In this experiment, when there is no nitrogen, the voltage is 0.398 V, and when pure nitrogen (99.999% purity) is introduced, the voltage is 0.347 V. From the calculation, we can get that the gas detected is nitrogen when the voltage drop variation is 0.051 V.

#### 3.2.3. Moisture Interference Test

**Figure 27:**
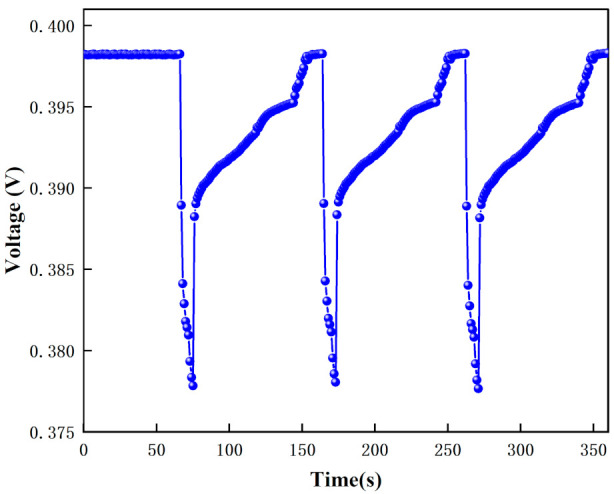
The test results of moisture.

In order to determine whether the moisture in human-exhaled gas has an effect on the test acetone, if there is an effect then the exhaled moisture should be filtered out by drying before the breath acetone test. The moisture (99.999% purity) was tested. The aeration volume for this experiment was the same as in Table 1. Figure 27 shows the test results of moisture. Figure 27 shows that the voltage decreases instantaneously at the moment of passing moisture; stop passing the moisture, the voltage will return to the initial value.This is due to the fact that the ionization energy of moisture is 12.6206 eV, which is greater than the energy of the UV lamp (10.6 eV), and the moisture cannot be ionized. When passing moisture, the small change in voltage is due to the change in pressure in the test chamber. Through the moisture voltage drop and through the acetone voltage remains unchanged or rises, excluding the impact of the moisture on the acetone measurement.

**Figure 28:**
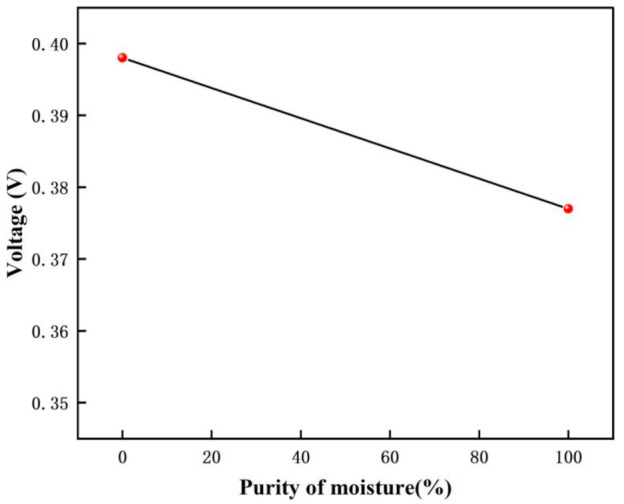
The voltage response graph for different purity moisture environments.

**Figure 29:**
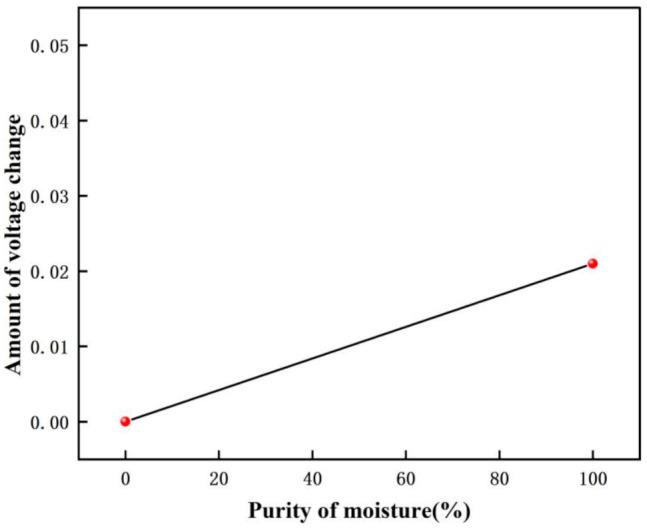
The voltage variation graph for different purity moisture environments.

The voltage response graph for different purity moisture environments are shown in Figure 28, and the voltage variation graph for different purity moisture environments are shown in Figure 29. In this experiment, the voltage is 0.398 V when there is no moisture, and 0.377 V when moisture (99.999% purity) is introduced. From the calculation, we can get that the gas detected is moisture when the amount of the voltage drop change is 0.021 V.

The above tests show that the sensor test nitrogen and moisture through the voltage drops slightly; the fluctuations are small, while the sensor test acetone voltage rises significantly and excludes the nitrogen and moisture on the acetone test interference, proving that the gas causing the sensor voltage rise is acetone.

We also compare the performance of the designed sensor with other acetone sensors. Table 2 shows the comparison of the present acetone sensor with the common acetone sensors.

The above experiments validate the performance of the FAIMS-MEMS-based acetone sensor, which can rapidly detect acetone at room temperature. The utilized FAIMS technology has the advantages of high sensitivity, fast response, good non-invasiveness, fast response time, simple structure, and a low detection limit. The sensor shows no change at 0.8 ppm acetone and a significant change at acetone concentrations above 1.8 ppm, which has a broad application prospect in the field of type 1 diabetes. In addition, we set a detection limit of 0.8 ppm acetone in this sensor, which can now be achieved with such a resolution. At a 0.8 ppm acetone detection the sensor does not change. More than a 0.8 ppm acetone detection voltage increased significantly, and the higher the acetone concentration, the greater the voltage change. The sensor is simple and clear, with a fast and convenient detection of acetone. In the future, we can improve the specific process of the sensor in order to reduce the detection limit of the sensor and determine whether a person has type 1 diabetes by the ratio of the voltage change of the acetone sensor exhaled by a person with type 1 diabetes to the voltage change of the acetone sensor exhaled by a normal person.

## 4. Summary

In this paper, a FAIMS-MEMS gas sensor with an integrated parallel migration zone collection area is designed. The sensitive structure of this sensor is processed by the MEMS process, and the sensor structure is simple, small, and easy to process. In the experiment, different concentrations of acetone were tested. The test results show that when 0.8 ppm of acetone is introduced, the voltage of the sensor basically does not change, while when 1.8 ppm of this gas is introduced, the voltage has a significant increase. The higher the concentration of acetone gas, the greater the voltage change; when 99.999% of pure nitrogen and moisture is introduced, the voltage of this sensor drops slightly, excluding the interference of nitrogen and moisture on acetone gas. The sensor is expected to be further developed in the field of non-invasive detection of type 1 diabetes because of its stability and repeatability, as well as its simple structure and small size. In conclusion, we have successfully prepared the FAIMS-MEMS gas sensor and completed the laboratory phase testing to propose the idea that the sensor can be applied to type 1 diabetes detection; however, it has not yet been put into the clinic. In the future, we will work with hospitals to verify if a patient has type 1 diabetes with our FAIMS-MEMS sensor, which we believe will have great potential in the field of type 1 diabetes.

## Figures and Tables

**Table 1:** The different gas concentrations corresponding to the different cycles in the experiments.

0–60 s	61–100 s	101–160 s	161–200 s	201–260 s	261–300 s	301–360 s
Air	0.8 ppm acetone	Air	0.8 ppm acetone	Air	0.8 ppm acetone	Air
Air	1.8 ppm acetone	Air	1.8 ppm acetone	Air	1.8 ppm acetone	Air
Air	10 ppm acetone	Air	10 ppm acetone	Air	10 ppm acetone	Air
Air	20 ppm acetone	Air	20 ppm acetone	Air	20 ppm acetone	Air
Air	Nitrogen	Air	Nitrogen	Air	Nitrogen	Air
Air	Moisture	Air	Moisture	Air	Moisture	Air

**Table 2:** The comparison of the present acetone sensor with the common acetone sensors.

Material	Type	Sensitivity	Detection Limit (ppm)	Operating Temperature (℃)	Reference
Si: WO3	Metal oxide gas sensors	4.3(S = Rair/R_acetone_ − 1)	0.02	400	[11]
In_2_O_3_	Metal oxide gas sensors	0.6%	25	400	[31]
ZnO	Metal oxide gas sensors	5.71%	100	200	[32]
ZnO + Ni +UV light	Metal oxide gas sensors	1.61%	100	RT	[33]
NiO-ZnO	Metal oxide gas sensors	−0.25(S = I_acetone_/I_air_ − 1)	0.11	RT	[34]
InN	Metal nitride gas sensors	28.7%	0.4	200	[35]
GaN	Metal nitride gas sensors	23%	500	350	[36]
Si: BF33, Au	FAIMS-MEMS	0.02 ppm/mV	0.8	RT	This work
